# Problem alcohol drinking in rural women of Telangana region, Andhra Pradesh

**DOI:** 10.4103/0019-5545.74309

**Published:** 2010

**Authors:** Padmavathy S. Potukuchi, Prasada G. Rao

**Affiliations:** Department of Psychiatry, Bhaskar Medical College and General Hospital, Moinabad, India

**Keywords:** Pregnancy, problem alcohol use, rural women, Telangana

## Abstract

**Background:**

This is the first ever study conducted to assess the prevalence of problem alcohol use in the rural women of Telangana region of Andhra Pradesh.

**Aims:**

To evaluate the prevalence of dependence and problem drinking, observe the factors that led to it and to monitor the effect of intervention in the form of psycho-education on their treatment seeking attitude.

**Materials and Methods:**

Cases were referred by the registrar from the Medicine Out-Patient Department using a three-item questionnaire for history of alcohol intake. Consecutive consenting female patients fulfilling the inclusion–exclusion criteria formed the sample. ICD-10 criteria and CAGE Questionnaire were used to assess dependence, problem drinking and co-morbid psychiatric illnesses. The socio-demographic data and the details regarding the nature and pattern of drinking and its complications were recorded using a semi-structured proforma. All patients were instructed to report at the end of 1 and 3 weeks for follow-up after a brief psycho-education regarding the problems of alcohol use.

**Results:**

Dependence was seen in 4.1% and problem drinking in 1%. Physical complications possibly due to alcohol were seen in 4.1% and psychiatric co-morbidity in 1%. Pregnancy drinking was recorded in 4.4%. Only 0.2% came for follow-up.

**Conclusion:**

To conclude, there is a perceptible degree of problematic use of alcohol in the rural women of this region. Yet, none of them were seeking psychiatric help. The soaring number of pregnancy drinking needs further exploration. The poor psychiatric follow-up leads us to conclude that in this sample the perception of alcohol problem is very low.

## INTRODUCTION

Alcohol use in women in India has been on the rise owing to the changing sociocultural milieu.[1] It is increasingly being recognized as a serious threat to their health and well being due to their unique, gender-based, physiological vulnerability factors.[2–5] However, there is a gross disparity in our knowledge about the problem alcohol use in Indian women for want of literature focusing exclusively on them. The dearth is more deeply felt for the rural sector, where, apart from urbanization, factors such as culture play a major role in the use of alcohol. The current prevalence of alcohol dependence in men, as quoted by a National Household Survey (2000–2001) is 21%.[6] On the other hand, the statistical data for women remain vague and unclear. It has been consistently reported to be less than 5%.[7] But the unrecorded consumption and expenditure on alcohol remains still higher. Hence, addressing this issue in the rural women, a morbidity which is reversible, especially from the rural areas, becomes the need of the hour. This study attempts to highlight the problem alcohol use among the rural Telangana women, where many unique cultural beliefs exist in the background.

When we look at some of the rural studies on alcohol in the last four decades, we see a predominant representation of male use.[8–13] A comprehensive analysis in both the genders, of the pattern of alcohol use and the socio-demographic details, was done in two phases in a rural study from Rajasthan in 1984.[14] To the best of our knowledge, the first ever detailed work-up on female alcohol use was done by a Bangalore based study (1994) where a definite fourfold increase in the female registries with problem alcohol drinking has been documented.[15] However, the women in the sample were mostly from the urban locale. A recent large epidemiological survey from Karnataka (2003) gives an elaborate account of female alcohol use in India, drawing the sample from both urban and rural districts. 58.6% of rural women in their sample were in the heavy drinking category compared to 40.4% of urban women. Overall, hazardous drinking was recorded in about 28% of women.[16] A retrospective analysis of substance abusing women from North India (2005) has reported alcohol as the second commonest drug of abuse in their sample in which the majority were urban.[17] To sum up, the above review brings to light the paucity of literature on rural female alcohol use despite the fact that heavy drinking is comparatively much higher among them. Based on our experience of attending on women with alcohol problems during the liaison with the medical speciality in our teaching hospital, which is located in a rural district of Telangana region, Andhra Pradesh, we started the work with the aim to identify the extent of the problem in the women of this region where Toddy use is culturally accepted. The dearth in the studies on problem alcohol drinking in rural women further motivated us to analyze the alcohol habit in this unexplored population. We also intended to observe the effect of intervention in the form of psycho-education regarding the problems of alcoholism. Telangana region of AP has 10 districts and has a population of about 24 million of which 73% lives in the rural areas.[18] Our medical college hospital offers predominantly secondary and tertiary level of care catering to a population of about 50,288 from 38 villages under 9 subcenters around a radius of about 100 km.

### Aims

To evaluate the prevalence of dependence and problem drinking among the rural women residing in the villages around our hospital, to observe the factors that led to dependence in this group and also to assess the role of brief intervention on their treatment seeking behavior.

## MATERIALS AND METHODS

A three-item screening questionnaire with yes/no answers to screen for alcohol intake ever in life to identify the cases was administered by the medical registrar in charge of the Medicine OPD (eliciting the current use, past use and presence of withdrawal symptoms). CAGE Questionnaire (1984)[19] (cutting down, annoyed by criticism, guilt about drinking and eye opener drink) and ICD-10[20] criteria were used for screening for dependence and problem drinking. ICD-10 criteria were also used for screening co-morbid psychiatric illnesses. A standardized scale for socioeconomic status[21] has been used to elicit the socio-demographic data. Using a semi-structured proforma, age at onset of drinking, nature, quantity and frequency of drinking (both at initiation and the current status), access to alcohol, causes for initiating and maintaining, duration of drinking, family history, other drugs abused, attempts at quitting in the past, the last drink, awareness about treatment, drinking during pregnancy and development of physical and psychological complications and co-morbidities were elicited. We have used the alcohol equivalents based on the percentage of alcohol by volume (ABV) in toddy (it contains 4–5% of ABV).[22]

### Inclusion–exclusion criteria

All successive female patients attending the medicine out-patient department (OPD) of our hospital with history of alcohol intake ever in life, from the rural areas around the hospital, who consented for the study were included.

Those who were critically ill, had cognitive impairment hampering the interview and were not willing were excluded.

### Method

At the initial screening, women with any one positive response in the three-item questionnaire were then referred for a psychiatrist’s evaluation. Sample was selected from the patients thus referred, and a detailed history and clinical features were recorded using the semi-structured proforma, CAGE Questionnaire and ICD-10 criteria. Psycho-education in the form of a brief orientation to alcohol related problems and its management and also the importance of abstinence on their improved physical and mental health was carried out for the group of patients selected at the end of the day. They were instructed to report back at the end of 1 and 3 weeks for follow-up.

### Sample

About 1400 women attended the medical OPD during the study period extending between March 2009 and June 2009. Seventy-one patients fulfilled the study criteria out of the 915 referred.

## RESULTS

Majority of the sample was above 50 years of age. Of this, a significant chunk belonged to average socioeconomic status (74.6%). Total illiteracy was seen in 87.3% of these women. All of them were married; nearly half of them were widowed [Table 1].

**Table 1:** Socio-demographic data

Age	
21–30 years	5 (7)
31–50 years	31 (43.6)
>51 years	35 (49.2)
Literacy (years of schooling)	
Nil	62 (87.3)
Up to 5	7 (9.8)
>5	2 (2.8)
Marital status	
All married	71 (100)
Widowed	33 (46.4)
Occupation	
Housewife	33 (46.4)
Laborer	33 (46.4)
Others	5 (7)
Religion	
Hindus	71 (100)
Socioeconomic status	
High	9 (12.6)
Medium	53 (74.6)
Low	9 (12.6)

Figures in parentheses indicates percentage

Toddy was the most abused alcoholic drink among this section of people (59%), though in 40.8%, multiple drinks in addition to toddy, such as beer and whisky were consumed at different times. The intake of alcohol started at the pediatric age group in about 39.4% of the cases. But the majority (49.2%) of the cases had their onset between 13 and 20 years of age. For those who tasted early in their life, it was first offered to them by either their parents or their grand parents as a refreshing beverage (71.8%). Daily drinking of one bottle of toddy (750 ml), approximately two drinks, was seen in 30.9%, though the preferred frequency was several times a week. Beer was usually consumed in social occasions along with toddy either once a month/several times a year during festivals/social get-togethers, the usual quantity quoted by most of them being ½–1 bottle. About 30–60 ml of whisky was consumed every day by 4.2% of the cases. Nearly 39.4% of them got alcohol by themselves, whereas 22.5% got through peers. Intake only during social occasions was seen in 1.4%. Yet, 9.8% had multiple sources of alcohol. The duration of alcohol intake in the screened cohort was not less than 30 years (49.2%). The duration of daily intake ranged from 3 months to 45 years. In this period, an increase in the frequency of alcohol intake was seen in 50.7%, the most common reason being sleeplessness. Other causes reported were stress, easy availability and financial freedom. Abstinence from toddy at the time of the interview was reported by 26.7%. The maximum duration of abstinence noted was 24 months in a subject. But other alcoholic beverages were consumed at social occasions. In this sample, about 68.4% reported physical ailments as the cause for abstinence. Factors maintaining continuous alcohol intake were withdrawal symptoms in 81.6% of the cases. As far as complications are concerned, 19.7% of them fulfilled ICD-10 criteria for problem drinking, 80.9% for dependence. Withdrawal delirium was seen in 11.2% of the cases. Of these, 37.5% consumed both toddy and whisky daily and the rest only toddy. Physical problems such as anorexia, gastritis and hypertension were seen in 80.9% of the cases. Depression was seen in 8.4% of the sample of which one had conversion reaction and another presented with suicidal attempt. A case of Generalized Anxiety Disorder was also registered [Table 2]. Pregnancy drinking was seen in 85.9% of the women studied. About 59.1% of them were advised to quit by different family members or doctors on various occasions. Lack of awareness about treatment availability for dependence was reported by about 97.1% of the sample. Family members including spouse, parents and siblings were habitual drinkers in 84.5% of the cohort. Though all patients were instructed for review after 1 week, only three of them came for follow-up, maintaining abstinence. They were put on supportive treatment in addition to counseling.

**Table 2:** Pattern of toddy drinking and complications seen in the sample

Quantity and frequency	22 (30.9)
Two drinks* - Daily	23 (32.3)
More than two drinks- Several times/week	
Age at initiation (years)	
<12	28 (39.4)
13–20	36 (50.7)
21–30	4 (5.6)
31–50	3 (4.2)
Maintaining factors	
Withdrawal symptoms	58 (81.6)
Aches	11 (15.4)
Cultural factors	5 (7)
Complications	
Physical	
Gastro-Intestinal symptoms and hypertension	55 (80.9)
Psychological	
Dependence	55 (80.9)
Problem drinking	14 (19.7)
Delirium	8 (11.2)
Co-morbidity	7(9.8)

* Two drinks = 750 ml of toddy (one bottle) containing 4–5% ABV[23]; Figures in parentheses indicates percentage

## DISCUSSION

In our study, the prevalence of chemical dependence and problem drinking was found to be 4.1 and 1%, respectively. Though this rate falls within the national figures, this is just the tip of the iceberg as the study sample is constituted by only 8% of the total number of cases referred after initial screening. It should be noted that 65% of the OP population screened has been consuming alcohol either in the past or present. This can only be explained possibly because culturally toddy is considered as a socially accepted drink. Although these figures may not be representative of the general population, it gives us a clear idea about its silent, but morbid, existence among the rural females in a surprisingly high degree.

### Extent and severity of the problem in the rural areas: Underreporting or overrepresentation?

Underreporting of female alcohol use appears to be a global phenomenon even in the wet cultures such as the west.[24] This may be because of the unique gender specific factors, sociocultural factors, economic and physician related factors. The stigma that is attached to alcoholism is the major stumbling block for the early screening in women, which is most often responsible for the under-diagnosis. In addition, the society’s denial of its existence and its consequences in them adds to the problem.[25] Even in our study, the gross disparity in the number that was identified and the number that presented themselves for the study could be explained by the same factors. Moreover, the women did not present themselves primarily for alcoholism at our psychiatry service. They rather sought medical help for the physical complications, possibly due to alcohol. This finding concurs with one of the previous studies where the authors pointed out co-morbid psychiatric conditions as the cause for seeking help in the majority rather than alcoholism *per se*.[15] Only 3% of the sample was referred by physicians for de-addiction in their study.

### Nature and pattern of drinking

The rural women of our sample preferred solitary drinking at home, usually in the evenings before their night meal. Beer and whisky were consumed more as a social drink to honor the guests. Here the women drank because it relieved the aches and made them fresh, gave good sleep, prepared them for the next day. Though a negligible number indulged in daytime drinking, no eye opener drink or any guilt about their drinking behavior was reported. Similarly, no binge drinking was seen in our sample. No aggressive or disinhibited behavior was reported in any of the women. Family involvement in the habit seems to be high in this population which possibly acts as a hindrance for abstinence. Widowed women with substantial financial freedom were found to be drinking more than one bottle of toddy along with whisky on a daily basis. Some differences could be drawn between our sample and the rural sample of the Karnataka study[16] in the pattern of drinking: the women in our sample started at a young age, initiated predominantly by a significant family member, more as a medicine or for recreation, the quantity usually not exceeding two drinks per occasion in the majority, presenting to the hospital mainly for seeking medical help for physical complications possibly secondary to alcohol intake. Marital life often remained undisturbed. Patients continued to drink a fixed quantity even after becoming dependent.

### Attitude of the patient toward treatment

In the initial interview, 97.1% of the study sample reported that they were unaware of the availability of treatment for withdrawal symptoms. Contrary to the expectation, only 4.2% of the sample (0.2% of the OP population) reported for one follow-up despite intervention in the form of brief psycho-education. This contradicting observation highlights the presence of other more serious and deep rooted problems hindering the patient from seeking treatment. The problems in the diagnosis and treatment of female alcoholism appears to be universal as follow-up failures are reported by studies from India[15,17] as well as the west.[24] What matters most is perhaps the gender and the societal expectations about its role. A Canadian study reported a number of barriers to treatment, which were found to be stronger for rural women compared to the urban.[24] Many of the barriers mentioned were perceived even in our sample: poor accessibility of health care providers, being one of the main bread winners of the family finding it difficult to commit for a long-term treatment, women’s health issues not given a serious thought as long as the person is functioning, illiteracy, failure to accept a long believed substance of recreation to be a source of problem, difficulty in procuring the drugs prescribed, peer pressure and poor co-operation from the family.

Until and unless these barriers are addressed, a complete treatment of alcohol abuse in rural females may not be possible.

### Special issues

#### Elderly drinking

About 9% of the OPD population was constituted by the elderly and 22% of the study sample belonged to the elderly category (above 60 years). Prevalence of dependence was found to be 13.8% in this group. A study from rural Haryana (2005) has recorded alcohol use in 0.8% of elderly women in their sample,[26] whereas at an all India level, 3.1% elderly females consume alcohol.[27] A rural Iowa study documented alcohol prevalence among their elderly women to be 41% where there was cultural acceptance.[28]

About 16.2% of the elderly were having gastrointestinal complaints and 11.3% had associated hypertension. Depression was seen in 2.4% and one had recently attempted suicide.

#### Other drugs of abuse in the elderly

Other drug use was minimal except for chewable tobacco in the form of zarda pan seen in 7.3%. Smoking was not seen in any of the females studied, whereas the Haryana study reported 41.1% of smoking women and only 5.4% of tobacco chewers.[26] This highlights the regional variations in the drug forms used.

#### Pregnancy drinking

Drinking during pregnancy was found to a high degree in this culture. In our sample, 85.9% of the women had taken alcohol during pregnancy with a positive expectancy about it. Of this, about 8% were first time drinkers who became dependent later on. The prevalence is estimated to be 4.3% as against a national figure of 2.3% of drinking during pregnancy as quoted by the National Family Health Survey 2005–2006.[7] The interesting finding is the cultural belief that toddy improves fetal movements, facilitates easy expulsion of placenta after delivery of the fetus and acts as a diuretic which is desired during pregnancy. Toddy was offered as soon as pregnancy was diagnosed. The quantity and frequency of intake varied from 200 to 750 ml, either twice a week or daily, depending upon their prior habituation. After delivery, toddy was not given as the woman started breast feeding. This feature is not discussed in most of the previous studies.

## CONCLUSION

To conclude, the analysis of a sample of rural women from the Telangana region of AP, attending the medicine OPD of a rural medical college hospital, for alcohol use, gave us the following insights. A considerable discrepancy in the prevalence of alcohol use that gets recorded and that actually exists among these women became evident. Unique factors leading to dependence were identified in this group. Though there is a discernible degree of dependence and problem drinking among these women, none reported at the psychiatry service for treatment of alcoholism. High rate of pregnancy drinking was observed which needs further exploration. Brief psycho-education intervention was attempted. But the follow-up for the psychiatric care being very low leads us to conclude that in this sample the perception of alcohol problem is very poor, which might be because of a high degree of social acceptance of alcohol use.
